# Differential expression of microRNAs during melanoma progression: miR-200c, miR-205 and miR-211 are downregulated in melanoma and act as tumour suppressors

**DOI:** 10.1038/bjc.2011.568

**Published:** 2012-01-05

**Authors:** Y Xu, T Brenn, E R S Brown, V Doherty, D W Melton

**Affiliations:** 1 MRC Institute of Genetics and Molecular Medicine, University of Edinburgh, MRC Human Genetics Unit, Western General Hospital, Crewe Road, Edinburgh, EH4 2XU, UK; 2 Department of Pathology, NHS Lothian, Western General Hospital, Crewe Road, Edinburgh, EH4 2XU, UK; 3 Edinburgh Cancer Centre, NHS Lothian, Western General Hospital, Crewe Road, Edinburgh, EH4 2XU, UK; 4 Department of Dermatology, NHS Lothian, Level 1, Lauriston Building, Lauriston Place, Edinburgh, EH3 9HA, UK

**Keywords:** melanocyte, malignant melanoma, naevus, invasion, anchorage-independent growth

## Abstract

**Background:**

The incidence of malignant melanoma is increasing faster than that for any other cancer. Histological examination of skin excision biopsies remains the standard method for melanoma diagnosis and prognosis. Significant morphological overlap between benign and malignant lesions complicates diagnosis, and tumour thickness is not always an accurate predictor of prognosis.

**Methods:**

To identify improved molecular markers to support histological examination, we used microarray analysis of formalin-fixed and paraffin-embedded samples from different stages of melanomagenesis to identify differentially expressed microRNAs (miRNAs). Differential expression was validated by qRT–PCR, and functional studies were carried out after transfection of miRNA precursors or inhibitors into melanoma cells to modulate miRNA expression.

**Results:**

In all, 20 miRNAs showed highly significant differential expression between benign naevi and either primary or metastatic melanomas, the majority being downregulated in melanoma, whereas only 2 miRNAs, namely miR-203 and miR-205, were differentially expressed between primary and metastatic melanomas. In functional *in vitro* assays, overexpression of miR-200c and miR-205 inhibited anchorage-independent colony formation and overexpression of miR-211 inhibited both anchorage-independent colony formation and invasion.

**Conclusion:**

We have identified a series of differentially expressed miRNAs that could be useful as diagnostic or prognostic markers for melanoma and have shown that three miRNAs (namely miR-200c, miR-205 and miR-211) act as tumour suppressors.

Globally, the incidence of malignant melanoma is increasing faster than that for any other cancer. In the United Kingdom, melanoma has become the second most common cancer among young adults (Cancer Research UK Cancer Statistics, 2011; http://info.cancerresearchuk.org/cancerstats/). Although some melanomas may show an unpredictable course, the histological measure of tumour thickness (Breslow thickness) remains the best predictor of outcome (Balch *et al*, 2001). However, it is not always an accurate indicator of biological behaviour. Although 5-year survival for patients with tumours <1 mm thick is >90%, a significant minority with thin melanomas go on to develop metastatic disease, whereas not all thick lesions metastasise. The mainstay of treatment is surgical, which may be curative for thin melanomas, emphasising the importance of early clinical detection and correct histological diagnosis. Histological examination of skin excision biopsies remains the diagnostic gold standard (Carlson *et al*, 2005); a challenging task because of the wide morphological spectrum of cutaneous melanocytic tumours and the lack of firm diagnostic criteria.

There is an urgent need for molecular markers to corroborate the diagnosis from histological examination. To be clinically useful, markers must be readily applicable to formalin-fixed and paraffin-embedded (FFPE) tissues and differences between benign and malignant lesions must be marked. There has been significant recent progress in understanding the genetic defects in melanoma, but few markers have proven to be of clinical significance and no single molecular marker has been informative over a wide range of lesion types (Carlson *et al*, 2005; Blokx *et al*, 2010).

MicroRNAs (miRNAs) are a large family of short non-coding RNAs that function as gene regulators. They can act as both oncogenes and tumour suppressors (Ventura and Jacks, 2009) and have advantages as melanoma biomarkers. Like mRNAs, they can be screened by array-based methods, but because of their very small size, they are not susceptible to degradation in FFPE samples. This is particularly important for melanoma in which the entire primary tumour is usually required for histology; hence, supply of fresh material for reliable mRNA microarray is severely limited.

Initial screening identified miRNAs in melanoma cell lines the expression of which was significantly different compared with other cancer cell lines (Blower *et al*, 2007; Gaur *et al*, 2007). MicroRNAs involved in melanomagenesis have now been identified from expression studies on fresh tissue and functional studies in cell lines (Mueller and Bosserhoff, 2009; Caramuta *et al*, 2010). Our approach has been to screen FFPE samples from different stages of melanomagenesis to identify differentially expressed miRNAs for functional studies, which have shown that three of these miRNAs act as tumour suppressors in malignant melanoma cells.

## Materials and methods

### Tissue samples

Formalin-fixed and paraffin-embedded samples were obtained from the Royal Infirmary of Edinburgh: benign naevi, recurrent and non-recurrent primary melanomas and metastatic melanomas. Details of the samples on the array are given in Supplementary Table 1. The local ethics committee granted approval for this study (REC reference number: 06/S1103/9). Samples of benign naevi and metastatic melanomas for RNA isolation were obtained from 2-mm diameter cores of FFPE blocks. For primary melanomas, tumour tissue was separated from adjacent non-tumour tissue by microdissection of 10-*μ*M sections. Suitable areas for coring and dissection were marked by the pathologist. RNA was isolated from FFPE samples (3–4 cores, or scrapings from 15 sections per isolation) using the RecoverAll Total Nucleic Acid Isolation Kit (Ambion, Life Technologies Ltd, Paisley, UK) following the manufacturer's protocol. Total RNA yields from FFPE samples were 0.4–9.0 *μ*g and A_260/280_ ratios ranged from 1.58 to 2.07.

### miRNA microarray

Illumina miRNA microarray (Illumina, San Diego, CA, USA; catalogue no. MI 501-1001, part no. 11297743) was carried out on 200 ng RNA extracted from FFPE and cultured cell samples. Analysis of the built-in controls using the Illumina BeadStudio application showed that array data were of good quality and established gene expression detection limits. To preserve as many differentially expressed miRNAs as possible, miRNA signals were not background subtracted. Instead, a filter (detection *P*-value <0.01 in at least 80% of samples) was applied. From a total of 1146 probes, 862 (75.2%) passed through the filter. Thereafter, log_2_-transformed data were normalised using the Quantile method and analysed using R version 2.8.1 software (www.r-project.org/). Hierarchical clustering was performed using Cluster 3.0 (http://bonsai.ims.u-tokyo.ac.jp/~mdehoon/software/cluster) software and the TreeView programme (http://jtreeview.sourceforge.net/) was used for interactive graphical analysis. City block distance (Manhattan distance) and complete linkage clustering methods were used to analyse log_2_-transformed data. Differentially expressed miRNAs on the array were identified using the Limma Bioconductor package for R (http://www.bioconductor.org/biocLite.R). miR-92 was chosen as internal control for the qRT–PCR assays because of its abundant and relatively constant expression in cell and FFPE samples. The average miR-92 expression signal for the different samples on the microarray is shown in Supplementary Figure 1.

### Mammalian cell culture

The origin of immortalised human melanocyte lines, Hermes 1 and Hermes 4a, has been described previously (Gray-Schopfer *et al*, 2006). Human malignant melanoma cell lines (A375, C32, G361 and WM115) were obtained from the European Collection of Cell Cultures (Salisbury, UK). HBL was obtained from Gentaur (GENTAUR Europe, Kampenhout, Belgium). EDMEL3 was isolated by Ewan Brown from a melanoma metastasis. A375 cells were maintained in Dulbecco's modified Eagle's medium (DMEM), supplemented with 10% fetal calf serum, 25 U ml^−1^ penicillin and 25 *μ*g ml^−1^ streptomycin. RNA was isolated using RNA-Bee (AMS Biotechnology (Europe) Ltd, Abingdon, UK).

### Transfection

RNA was mixed with the siPORT NeoFX Transfection Agent (Ambion), and reverse transfection of A375 cells was carried out following the manufacturer's protocol. Cell number per well: 1 × 10^5^ for 12-well and 2 × 10^5^ for 6-well plates. Transfection efficiencies for precursor- and anti-miRNAs were first optimised using FAM-labelled pre-miR and anti-miR negative controls (Ambion), the uptake of which into cells was monitored by flow cytometry. Final concentration of RNA and transfection agent: RNA, 30 nM for precursors, 150 nM for inhibitors; siPORT NeoFX, 8 *μ*l per well in 12-well plates, 16 *μ*l per well in 6-well plates. These conditions consistently yielded transfection efficiencies >85% with A375 cells.

### Gene expression assays

Both miRNA and mRNA expressions were determined by TaqMan assays. Assays for individual miRNAs and mRNAs, reverse transcription and PCR kits were all obtained from Applied Biosystems (Carlsbad, CA, USA). The miRNA expression was standardised against the miR-92 control and mRNAs were standardised against *β*-actin. A standard curve was run for each gene-specific PCR reaction to determine whether the PCR efficiencies of target and control gene were equivalent. In every case, the slope of the plot of log dilution factor *vs* ΔC_T_ was <0.1; hence, the comparative C_T_ method could be used to determine relative miRNA and mRNA concentrations (Livak and Schmittgen, 2001).

### Proliferation and cell-cycle distribution assays

Growth rate was determined using the sulforhodamine B colorimetric growth assay (Vichai and Kirtikara, 2006). Cell-cycle distribution was determined by flow cytometry of propidium iodide-stained nuclei.

### Methylcellulose colony-formation assay

Cells (1 × 10^4^) were added in 2 ml of 1.4% methylcellulose in complete DMEM onto a 2 ml layer of 1.8% agarose in complete DMEM in 6-well tissue culture plates. After incubation, colony size was determined by image analysis of microscopic images.

### Invasion assay

The transwell migration assay was carried out as described previously (Serrels *et al*, 2010). After incubation, cells were dye-labelled and visualised in the Matrigel basement membrane matrix (BD Biosciences, Oxford, UK; Cat. no. 354 234) at 10-*μ*M intervals by confocal sectioning. The relative cell number in each section, determined from fluorescence intensity, was analysed using ImageJ software (NIH, http://rsb.info.nih.gov/ij/) and expressed relative to the cell number in the section that represented the base of the transwell filter. Significance tests for the results of this and the other cell-based assays were carried out using one-way ANOVA or Student's *t*-test.

## Results

### Hierarchical clustering of miRNA expression patterns from FFPE and cultured cell samples

MicroRNA microarray expression data were obtained from 52 FFPE specimens (11 benign naevi, 10 recurrent and 10 non-recurrent primary melanomas, 21 metastatic melanomas) and 15 cell lines. The cell lines were 2 immortalised human melanocytes and 13 human malignant melanoma lines (A375, C32, G361, WM115, HBL and the EDMEL3 series). EDMEL3 was isolated from a late-recurring distal melanoma metastasis. Early and later passages, as well as subclones with different morphologies (epithelial, spindle cell, mixed) were available. Cells retrieved back into culture from EDMEL3 xenografts were also included. Further details of the samples on the array are given in Supplementary Table 1. Unsupervised cluster analysis was carried out on the entire data set. The clustering obtained along with part of the heatmap is shown in Supplementary Figure 2 to illustrate the EDMEL3 cluster, which has high expression for the 20 miRNAs selected compared with most of the other samples. The similarity between the metastasis, from which the EDMEL3 cell line was derived, and the EDMEL3 cell line series is illustrated in Supplementary Figure 2. However, all the melanoma cell lines clustered separately from the FFPE samples, stressing the importance of not relying entirely on cell culture data for studies on melanomagenesis. For FFPE samples, there was no clear clustering distinction between naevi, primaries and metastases, perhaps reflecting the molecular heterogeneity of melanoma. The pattern of clustering of FFPE samples was not affected when the melanoma and melanocyte cell data were excluded from the analysis. However, significant differences between the groups in the expression of individual miRNAs were found as described below.

### Melanoma-specific miRNA expression profile

The 735 target miRNAs on the array included 470 well-annotated human miRNAs and 265 additional miRNAs. The expression of each was compared individually between the naevus, primary and metastatic melanoma groups to identify melanoma-specific miRNAs. In all, 113 miRNAs were differentially expressed in the comparison between primary melanomas and benign naevi (adjusted *P*-value <0.05). In all, 97 miRNAs were differentially expressed between metastatic melanomas and benign naevi. In the comparison between metastatic and primary melanomas, only 25 miRNAs were differentially expressed. The full lists of the differentially expressed miRNAs are shown in Supplementary Table 2.

**Figure 1:**
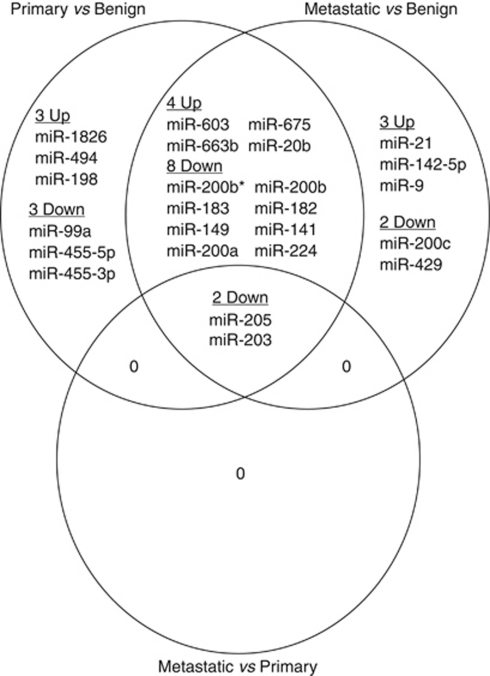
Top differentially expressed microRNAs between benign naevi, primary and metastatic melanoma. Venn diagram showing the relationship between the differentially expressed miRNAs from the comparisons shown in Table 1. For each X *vs* Y comparison, up means expression was higher in X, down means expression was lower in X. Naevus group, *n*=11; primary group, *n*=20; metastasis group, *n*=21.

There were too many differentially expressed miRNAs for all to be investigated further. To narrow the candidate list, more stringent criteria were added (log_2_ fold expression change >2 and adjusted *P*-value <0.001). Overall, 20 miRNAs remained in the primary melanoma *vs* naevus comparison, 19 in the metastatic melanoma *vs* naevus comparison and only 2 in the metastatic *vs* primary comparison (Table 1). There was considerable overlap between the three lists (Figure 1).

Only two miRNAs, miR-205 and miR-203, were present in all three comparisons and both were downregulated in melanomas. Their expression was decreased from the naevus through the primary melanoma to the metastatic melanoma group. All five members of the miR-200 family, miR-200a, miR-200b, miR-200c and miR-141 and miR-429 were decreased in the metastatic melanoma *vs* benign naevus comparison and three members (miR-200a, miR-200b and miR-141) were also downregulated in primary melanomas compared with benign naevi. Expression of miR-20b and miR-675 was increased in primary and metastatic melanomas in comparison with benign naevi. miR-211 was downregulated in metastatic melanomas relative to benign naevi (adjusted *P*-value 0.0087, log_2_ fold expression change 2.4), but did not pass through the stringent filter. It was also chosen for qRT–PCR verification because of the large expression difference found between melanocyte and melanoma cell lines (see later).

### Verification of differential miRNA expression during melanomagenesis

**Figure 2:**
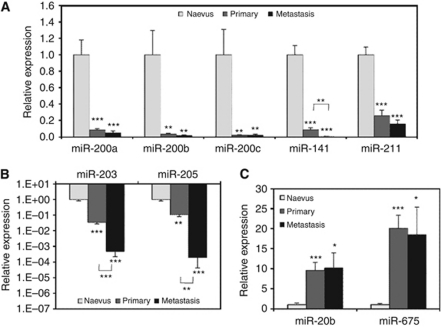
microRNA expression changes during melanoma progression. (**A**) Downregulated miRNAs miR-200a, miR-200b, miR-200c, miR-141 and miR-211. (**B**) Downregulated miRNAs miR-203 and miR-205. It must be noted that the *Y* axis is on a log_10_ scale. (**C**) Upregulated miRNAs miR-20b and miR-675. Mean expression (±s.e.m.) of each miRNA, determined by qRT–PCR, is shown relative to miR-92 and normalised to the Naevus group mean. Naevus group, *n*=12–17; primary, *n*=20; metastasis, *n*=14. ^*^P<0.05; ^**^P<0.01; ^***^P<0.001.

An additional eight benign naevi were included in the verification experiments. For every miRNA examined, the qRT–PCR data verified the microarray result. The four members of the miR-200 family (miR-200a, miR-200b, miR-200c and miR-141) showed significant decreased expression between benign and malignant tissues from 15- to 200-fold (Figure 2A). However, only miR-141 also showed a significant decrease between primary and metastatic melanomas. The expression of miR-211 was decreased in both primary (4-fold) and metastatic melanomas (6-fold) compared with benign naevi (*P*<0.001), but again there was no significant difference between primaries and metastases. For miR-203 and miR-205, there was a >10-fold decrease from benign naevi to primary melanomas and a >100-fold decrease from primary to metastatic melanomas. All expression differences between groups were highly significant (Figure 2B). The expression was 10-fold higher in melanomas compared to benign naevi for miR-20b and 20-fold higher for miR-675, although in each case, there was no significant difference between primary and metastatic melanomas (Figure 2C).

Although all nine miRNAs showed the same trend when comparing the non-recurrent and recurrent primary groups as observed in the primary with metastasis comparison, none of the differences were significant (Supplementary Figure 3).

### Overexpression of miR-200c in melanoma cells causes increased expression of E-cadherin mRNA

Both miR-200c and miR-205 were selected for functional studies because both act as tumour suppressors in other cancers by reversing epithelial-mesenchymal transition (EMT), in which one of the key events is reduction in E-cadherin levels. E-cadherin expression is controlled by the ZEB1 and ZEB2 transcriptional repressors, which are targets of both miR-200c and miR-205 (Maragkakis *et al*, 2009).

**Figure 3:**
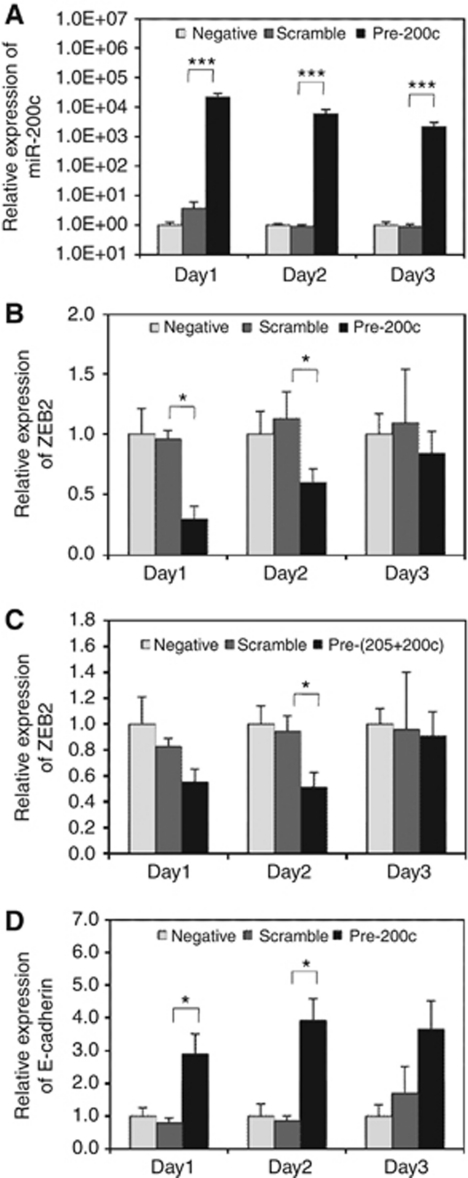
Ectopic expression of miR-200c in melanoma cells causes ZEB2 downregulation and E-cadherin upregulation. Expression was determined by qRT–PCR after transfection of A375 melanoma cells with miR-200c precursor, or a combination of miR-200c and miR-205 precursors (dark shading), or a scrambled control miRNA (intermediate shading); non-transfected negative control (light shading). Mean relative expression levels (±s.e.m.) from three independent experiments are shown (**A**) Expression of miR-200c relative to miR-92 and normalised to the mean of the negative control. (**B**) Expression of ZEB2 mRNA after transfection with miR-200c precursor relative to *β*-actin and normalised to the mean of the negative control. (**C**) Expression of ZEB2 mRNA after transfection with miR-200c and miR-205 precursors relative to *β*-actin. (**D**) Expression of E-cadherin mRNA after transfection with miR-200c precursor relative to *β*-actin. ^*^P<0.05; ^***^P<0.001.

The expression of miR-200c and miR-205 was significantly reduced in melanomas compared to benign naevi. The expression of both miRNAs was also low in the human malignant melanoma cell line, A375 (data not shown). For functional studies, precursors of miR-200c and miR-205 were transfected into A375 cells and expression was assayed by qRT–PCR. Levels of both miR-200c (Figure 3A) and miR-205 (Supplementary Figure 4A) were dramatically increased in precursor-transfected cells compared with non-transfected negative control and transfected scramble control groups. Levels were highest at 24h (>10^4^-fold increase for miR-200c) and then dropped over the time course.

The expression of ZEB2 was downregulated in the pre-200c group on all 3 days (Figure 3B). The reduction was significant on days 1 (4.5-fold) and 2 (2-fold) compared with the scrambled control, indicating that the inhibitory effect of miR-200c reduced over the time course as its level decayed. The expression of ZEB2 was also reduced in the pre-205 group on day 1, but the reduction was not significant (Supplementary Figure 4B). The expression of ZEB2 was also decreased on days 1 and 2 in cells transfected with both precursors, the decrease being significant on day 2 (Figure 3C).

There was no indication of an additive effect of miR-200c and miR-205 on the reduction of ZEB2 mRNA levels; hence, expression levels of the transcriptional target of ZEB2, E-cadherin, were only assayed in A375 cells with elevated miR-200c levels. E-cadherin mRNA levels were increased on all 3 days and the elevation was significant on days 1 and 2 (3- and 4-fold, respectively; Figure 3D), compatible with the reduction in ZEB2 mRNA observed on the same days.

### Overexpression of miR-200c and miR-205 in melanoma cells causes reduced anchorage-independent colony formation

**Figure 4:**
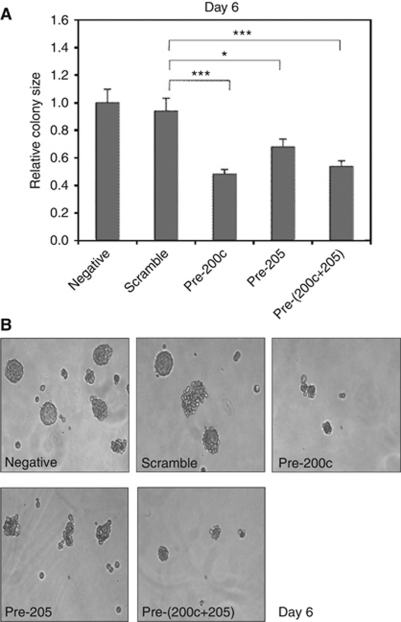
Ectopic expression of miR-200c in melanoma cells causes reduced anchorage-independent colony formation. A375 cells were transfected with miR-200c precursor, or a combination of miR-200c and miR-205 precursors, or a scrambled control miRNA and colony formation in methylcellulose was determined after 6 days. Non-transfected A375 cells were used as a negative control. (**A**) Histogram showing the mean size (±s.e.m.) of 30 colonies from each group, normalised to the mean of the negative control group. ^*^P<0.05; ^***^P<0.001. (**B**) Representative colony images on day 6.

A375 cells with elevated miR-200c and miR-205 levels growing in control medium had the same proliferation rate and cell-cycle distribution for 4 days after transfection as controls (data not shown). We next investigated the effect of elevated levels of miR-200c and miR-205 on tumourigenicity and invasion assays. Anchorage-independent colony formation in methylcellulose is a powerful *in vitro* surrogate assay for tumourigenicity in xenograft assays. A375 cells were set up in methylcellulose immediately after transfection with miR-200c and miR-205 precursors and with a control scrambled miRNA. Colony size on day 6 was significantly smaller for the miR-200c and miR-205 precursor-transfected groups, but again there was no indication of an additive effect with both precursors (Figure 4).

To determine whether elevated levels of miR-200c and miR-205 influence the invasive ability of A375 melanoma cells, a transwell migration assay through Matrigel was set up immediately after transfection and analysed on day 5 (Supplementary Figure 5). For the miR-200c and miR-205 precursor-transfected groups, the relative cell numbers 40 *μ*M into the Matrigel appeared lower than in the scramble and negative controls, but the differences were not significant.

### Overexpression of miR-211 in melanoma cells causes both reduced anchorage-independent colony formation and invasiveness

**Figure 5:**
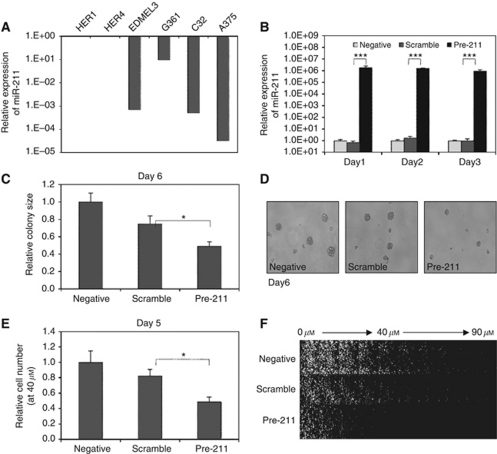
Ectopic expression of miR-211 in melanoma cells causes reduced anchorage-independent colony formation and invasion. A375 cells were transfected with miR-211 precursor, or a scrambled control miRNA and colony formation in methylcellulose and invasion in a transwell migration assay was determined. (**A**) Expression of miR-211 in melanoma and melanocyte cell lines. Immortalised melanocyte lines Hermes 1 (HER1) and Hermes 4a (HER4); melanoma lines EDMEL3, G361, C32 and A375. Mean miR-211 expression from two independent experiments relative to miR-92 and normalised to HER1 is shown. (**B**) Overexpression of miR-211 in A375 melanoma cells after transfection of precursor miR-211. Mean relative expression levels (±s.e.m.) of miR-211 relative to miR-92 and normalised to the mean of the negative control from three independent experiments are shown. (**C**) Reduced methylcellulose colony formation in A375 cells after transfection of miR-211 precursor. Histogram shows the mean size (±s.e.m.) of 30 colonies from each group on day 6, normalised to the mean of the negative control group. (**D**) Representative colony images on day 6. (**E**) Reduced invasion in A375 cells after transfection of miR-211 precursor. Cells migrating through Matrigel in a transwell inverse invasion assay were quantified after 5 days at the 40 *μ*M layer. The relative cell number was determined as the ratio of cell number in the 40 *μ*M layer/cell number in the origin (0 *μ*M layer). Results represent average ratios (±s.e.m.) of six microscope fields for each group, normalised to the negative control group. (**F**) Representative stacked confocal images from the origin (0 *μ*M layer) to the tenth layer (90 *μ*M). ^*^P<0.05; ^***^P<0.001.

The expression of miR-211 was >10-folder lower in all four melanoma cell lines examined than in the two immortalised melanocyte lines, and in A375, the expression was 10^4^-fold lower (Figure 5A). When the miR-211 precursor was transfected into A375 cells, the expression was dramatically increased on all 3 days after transfection, increasing levels to those seen in immortalised melanocytes (Figure 5B).

As with miR-200c and miR-205 overexpression, there were no obvious changes in proliferation rate or cell-cycle distribution in A375 cells with elevated miR-211 levels growing in control medium; hence, we proceeded to anchorage-independent colony-formation and invasion assays. Colony size in methylcellulose, determined on day 6, was significantly smaller for miR-211 precursor-transfected groups than for both scrambled and non-transfected controls (Figure 5C and D). Migration through Matrigel in the transwell migration assay was also significantly reduced on day 5 for miR-211 precursor-transfected cells (Figure 5E and F).

### Reduction of miR-20b has no effect on melanoma cells

The expression of miR-20b was significantly increased in melanomas compared with benign naevi. A miR-20b inhibitor miRNA was transfected into A375 cells so that the effects of decreased miR-20b levels could be studied. Expression was significantly decreased at 24 and 48h (5- and 2-fold respectively; Supplementary Table 3). The reduction for miR-20b was much less than the increases observed for miR-200c, miR-205 and miR-211 and the effect diminished more rapidly after transfection. Perhaps unsurprisingly, no effect of miR-20b reduction in A375 cells was observed in cell proliferation, cell-cycle distribution, anchorage-independent colony-formation and invasion assays (data not shown).

## Discussion

As an initial step to identifying improved diagnostic and prognostic molecular markers for melanoma, we carried out a comprehensive microarray study incorporating fixed tissues samples representing the key stages in melanomagenesis and a range of melanocyte and melanoma cell lines. As such, we believe it to be the largest and most complete study of miRNA expression in melanoma so far.

Melanoma cell lines did not cluster together with FFPE melanoma tissues, questioning the validity of many of the melanoma miRNA expression studies so far, which have been carried out exclusively on cell lines. The naevus, primary and metastatic FFPE samples were not found in discrete separate clusters, but were instead intermingled. A similar intermingling of melanocyte, primary and metastatic melanoma cell lines has been reported previously (Mueller *et al*, 2009), and we conclude that cluster analysis of miRNA expression does not distinguish the different stages of melanomagenesis.

Analysis of differential expression during melanomagenesis of individual miRNAs was more informative. In all, 20 miRNAs passed through a very strict filter for differential expression between benign naevi and primary or metastatic melanoma, and only 2, miR-203 and miR-205, passed through the same filter for differential expression between primary and metastatic melanoma. Nine of these miRNAs were selected for qRT–PCR validation and all passed the test. The majority of the differentially expressed miRNAs identified were downregulated during melanomagenesis. All members of the miR-200 family examined showed a 10-fold reduction from benign naevi to primary melanoma, but in only one, miR-141, was there a further significant reduction from primaries to metastases. The largest differential downregulation was shown by miR-203 and miR-205, in which expression was 10-fold lower in primaries than in naevi, and was then reduced a further 100-fold from primaries to metastases. Both miR-20b and miR-675 were two of the minority of miRNAs that were upregulated during progression from benign naevi to primary melanoma, but again with no increase on metastasis.

Firm diagnostic criteria for melanoma based on histological features alone are difficult to establish owing to the wide morphological range of melanocytic naevi and significant morphological overlap between benign lesions and malignant melanoma. There can be frequent disagreement on diagnosis, even between expert dermatopathologists (Grant-Kels *et al*, 1999; Lodha *et al*, 2008), and this could result in possible overtreatment and in delayed treatment. If detection by *in situ* hybridisation could be established for one or more of the most highly differentially expressed miRNAs between benign and malignant melanocytic lesions that we have identified, this could prove a very valuable diagnostic tool. Indeed, this has now been reported for miR-21 and miR-155, which were overexpressed in melanoma and in borderline melanocytic lesions (Grignol *et al*, 2011). Both of these miRNAs appear in our full list of miRNAs overexpressed in metastatic melanoma.

During the course of this study, a number of other melanoma-specific miRNA expression patterns have been reported, mostly from microarray studies on cultured cells and by tissue analysis, rather than from full microarray on FFPE tissues as in our study (Blower *et al*, 2007; Gaur *et al*, 2007; Mueller *et al*, 2009). Many of the miRNAs identified also appear in our lists. A study comparing 8 benign naevi and 8 metastatic melanomas, but no primary melanomas, described 31 differentially expressed miRNAs (Chen *et al*, 2010). Another study included three benign naevi, five primary and seven metastatic melanoma samples (Philippidou *et al*, 2010). Comparing these two reports, which used fewer FFPE samples, with our array results, some common differentially expressed miRNAs emerge: miR-200 family, miR-20b, miR-125b, miR-183, miR-193b, miR-203, miR-204, miR-205, miR-211, let-7c.

Although all nine miRNAs that we validated by qRT–PCR showed the same trend when comparing non-recurrent and recurrent primary melanomas as observed in the primary with metastasis comparison, none of the differences were significant. Larger sample numbers and actual length of survival comparisons, rather than simple recurrence status, would be required to investigate the prognostic value of these miRNAs. Two studies have reported prognostic miRNA expression profiles (Caramuta *et al*, 2010; Segura *et al*, 2010); of 18 such miRNAs in Segura *et al* (2010), 7 appear in our lists.

The reduced expression we observed for all five members of the miRNA-200 family in melanomas compared with benign naevi is consistent with previous reports (Chen *et al*, 2010; Philippidou *et al*, 2010). However, other studies have found one of more miRNA-200 family members upregulated in melanoma (Schultz *et al*, 2008; Mueller *et al*, 2009). miR-205 showed the largest reduction in expression during melanomagenesis, again consistent with a recent report (Dar *et al*, 2011). The miR-200 family and miR-205 reverse the EMT process required for metastasis by directly targeting the 3′ untranslated regions of the mRNAs for ZEB1 and ZEB2, which are transcriptional repressors of the E-cadherin gene in epithelial cancers (Hurteau *et al*, 2007; Bracken *et al*, 2008; Burk *et al*, 2008; Korpal *et al*, 2008; Park *et al*, 2008; Gregory *et al*, 2008a, 2008b). Epithelial-mesenchymal transition also appears to be important in a non-classical epithelium-derived cancer like melanoma (Park *et al*, 2008). Study of melanoma tissues suggested that EMT-related genes contributed to the promotion of the metastatic phenotype (Alonso *et al*, 2007). Suppression of melanoma cell growth *in vitro* and in xenografts and induction of senescence by overexpression of miR-205 has recently been reported (Dar *et al*, 2011). There is also now one report that miR-200a and miR-200c, although not suppressing melanoma cell invasion, had different effects on the mode of invasion (Elson-Schwab *et al*, 2010).

We chose miR-200c and miR-205 for our functional study in A375 melanoma cells and found that transient upregulation caused reduced anchorage-independent colony formation in an *in vitro* surrogate assay for tumourigenicity. There was no additive effect when both miRNAs were elevated together and no effect on invasiveness was observed in transwell migration assays. For miR-200c, this was accompanied by a reduction in levels of the ZEB2 target and an increase in its target, E-cadherin. Thus, our data demonstrate that miR-200c acts as a tumour suppressor in melanoma, the same role that has been attributed to the miR-200 family in other cancers, and confirm the role of miR-205 as a tumour suppressor in melanoma. Demonstration of stronger tumour suppressor and target pathway effects would require stable, rather than transient increased expression.

miR-211 showed significantly reduced expression in melanomas compared with naevi, but was mainly chosen for functional studies because of the 1000-fold decreased expression we observed in most of the melanoma cells lines examined compared with immortalised melanocytes. Reduced expression of miR-211 in melanoma compared with melanocyte cell lines (Caramuta *et al*, 2010; Boyle *et al*, 2011), in an invasive melanoma cell line compared with a less invasive derivative (Mueller *et al*, 2009), in primary melanomas compared with benign naevi (Chen *et al*, 2010; Jukic *et al*, 2010) and in melanomas that have spread to sentinel lymph nodes compared with those that have not (Glud *et al*, 2010) has been reported previously. Transient ectopic expression of miR-211 in melanoma cells significantly decreased both anchorage-independent colony formation and invasiveness. Reports published since our study began have also demonstrated the tumour-suppressor ability of miR-211 in invasion assays only in melanoma cell lines and have identified a number of targets, including transforming growth factor receptor II and the BRN2 transcription factor, which represses expression of MITF, the master transcription factor regulator for melanocytes (Levy *et al*, 2010; Mazar *et al*, 2010; Boyle *et al*, 2011).

miR-20b was chosen for functional studies because it was one of the minority of miRNAs that showed highly significant increased expression in melanomas compared with naevi. It modulates c-MYC and acts as an oncogene in human T-cell leukaemia and mouse mammary cancers (Landais *et al*, 2007; Sun *et al*, 2009). Transfection with an anti-miR was used to transiently knockdown miR-20b levels in melanoma cells, but no changes in anchorage-independent colony formation or invasiveness were found, possibly because of the small reduction achieved, particularly when compared with the large increases obtained for miR-200c, miR-205 and miR-211. An alternative explanation could be that miR-20b functions in carcinogenic processes, such as tumour angiogenesis, that we did not assay for (Hua *et al*, 2006; Lei *et al*, 2009).

In conclusion, we have carried out a comprehensive microarray analysis on naevi and melanoma samples to identify and validate miRNAs that are differentially expressed during melanomagenesis. Such miRNAs could serve as markers to improve melanoma diagnosis and prognosis. Functional assays on selected differentially expressed miRNAs in cultured melanoma cells have identified miR-200c and miR-205 as tumour suppressors in anchorage-independent colony-formation assays and miR-211 as a tumour suppressor in both anchorage-independent colony-formation and invasion assays.

## Figures and Tables

**Table 1:** Top differentially expressed microRNAs in benign naevi, primary and metastatic melanoma FFPE tissues

**Primary *vs* naevus**			**Metastatic *vs* naevus**			**Metastatic *vs* primary**		
**MiR**	**Adj. *P***	**Exprn**	**miR**	**Adj. *P***	**Exprn**	**miR**	**Adj. *P***	**Exprn**
603	1.45E-12	6.9	205	3.88E-16	−37.5	205	4.42E-09	−6.9
663b	7.04E-10	10.8	203	2.06E-15	−64.0	203	4.42E-09	−10.4
1826	8.69E-10	4.7	183	6.41E-11	−11.5			
200b^*^	1.26E-09	−11.5	200b^*^	1.27E-10	−13.7			
183	2.47E-09	−8.4	200c	1.27E-10	−8.8			
149	5.13E-07	−5.3	603	1.26E-09	4.6			
200a	1.55E-06	−6.9	200b	8.86E-08	−12.7			
205	2.49E-06	−5.4	200a	8.86E-08	−8.6			
675	4.87E-06	8.3	663b	9.07E-08	7.0			
99a	6.97E-06	−4.1	141	2.24E-06	−7.6			
200b	6.97E-06	−8.2	149	4.38E-06	−4.4			
455-5p	8.39E-06	−5.5	429	1.91E-05	−5.8			
182	1.76E-05	−7.1	21	2.42E-05	4.3			
203	2.91E-05	−6.1	224	3.06E-05	−8.9			
494	4.99E-05	7.5	182	3.06E-05	−6.5			
455-3p	1.93E-04	−4.4	142-5p	1.02E-04	4.8			
20b	2.55E-04	5.4	20b	2.26E-04	5.4			
141	5.14E-04	−4.5	675	2.90E-04	5.3			
198	7.98E-04	5.5	9	5.51E-04	7.4			
224	9.86E-04	−5.8						

Abbreviations: Adj. *P*=adjusted *P*-value; Exprn=expression difference; FFPE=formalin fixed and paraffin embedded.

Differentially expressed miRNAs with an Exprn >±4-fold and an Adj. *P* <0.001 are shown, ranked by adjusted *P*-value. In X *vs* Y comparison, when Y>X, the expression fold change is negative.
