# Supplementary material for: Differential expression of microRNAs during melanoma progression: miR-200c, miR-205 and miR-211 are downregulated in melanoma and act as tumour suppressors
Source: Br J Cancer. 2012 Jan 5;106(3):553–61. doi: 10.1038/bjc.2011.568 (PMC3273359; doi:10.1038/bjc.2011.568)
Supplement: Supplementary Information [file bjc2011568x1.doc]

**Supplementary Figure 1**


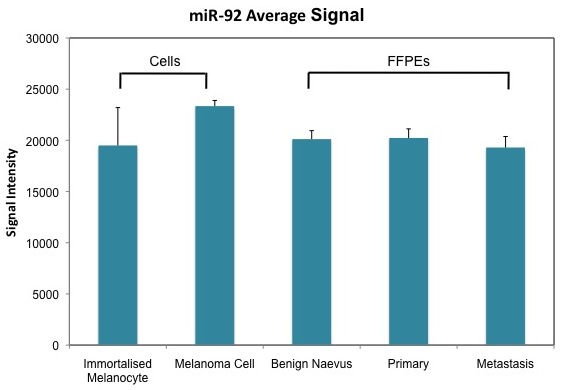


**Supplementary Figure 1** Average signal intensities for the miR-92 internal control of all groups of cell and tissue samples on the microarray. The histogram shows the average miR-92 fluorescent signal intensities (± SEM) for all sample groups.

**Supplementary Figure 2**

**
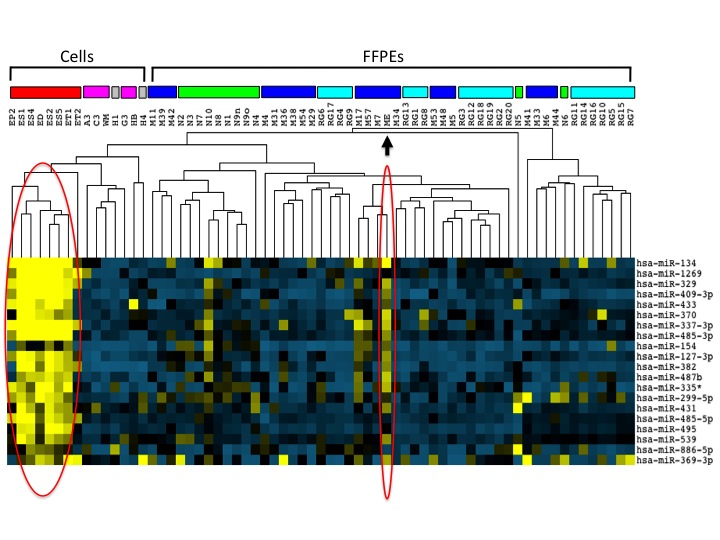
**


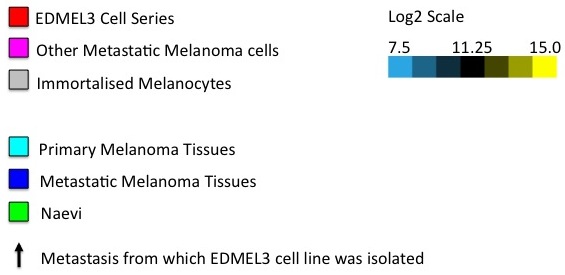


**Supplementary Figure 2** Hierarchical clustering of microRNA expression patterns. The clustering obtained for all 67 samples (15 cell lines and 52 FFPE specimens) using all miRNAs on the array that were expressed above the threshold level is shown. A colour key is shown above each sample to identify the type of cell line or tissue sample. The heatmap for 20 selected miRNAs is shown vertically below each sample to illustrate the clustering. The red ellipses illustrate the similarity between the different members of the EDMEL3 melanoma cell line series and the metastasis from which the EDMEL3 cell line was derived. Keys: coloured squares and a black arrow identify the type of sample; the log_2_ blue-yellow colour scale (7.5-15.0) depicts the log_2_ expression level of the miRNAs on the heatmap.

**Supplementary Figure 3**


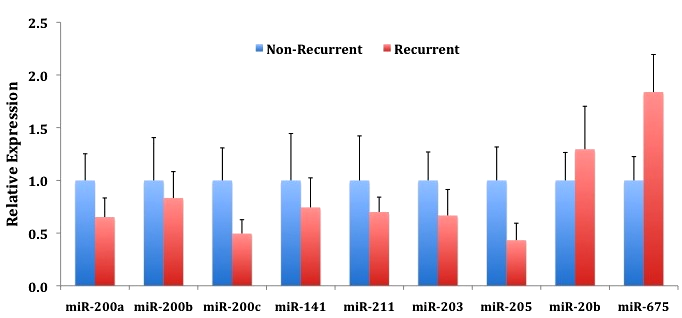


**Supplementary Figure 3** Comparison of microRNA expression levels between non-recurrent and recurrent primary melanoma samples. Mean expression (± SEM) determined by qRT-PCR of each miRNA relative to miR-92 and normalized to the mean of the Non-Recurrent group is shown. Blue bars, Non-Recurrent, n=10; Red bars, Recurrent, n=10.

**Supplementary Figure 4**

**A** Expression of miR-205

|  | **Negative** | **Scramble** | **pre-205** |
| --- | --- | --- | --- |
| **24 hr** | 1.00±0.48 | 1.42±0.51 | 1392245±552660 |
| **48 hr** | 1.00±0.56 | 0.97±0.10 | 521964±142580 |
| **72 hr** | 1.00±0.34 | 1.50±0.59 | 179269±61807 |

**B**


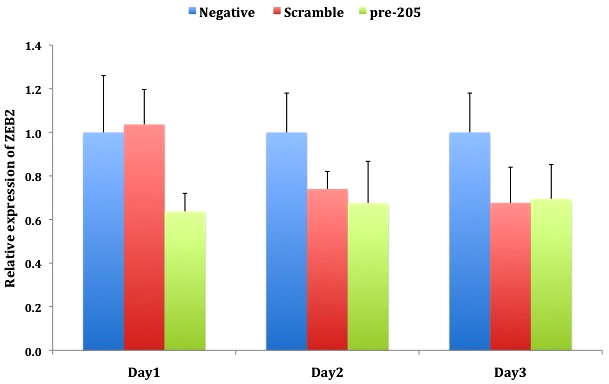


**Supplementary Figure 4** Ectopic expression of miR-205 in cultured melanoma cells fails to cause downregulation of ZEB2. A375 melanoma cells were transfected with miR-205 precursor, or a scrambled control miRNA. (**A**) Mean relative expression levels of miR-205 (± SEM), relative to miR-92 and normalized to the mean of the negative control from three independent experiments, are shown for the three days following transfection. (**B**) Mean relative expression levels of ZEB2 (± SEM), relative to beta-actin and normalized to the mean of the negative control, are shown. Blue bars, non-transfected negative control; red bars, scrambled control miRNA; green bars, miR-205 precursor.

**Supplementary Figure 5**

**A**


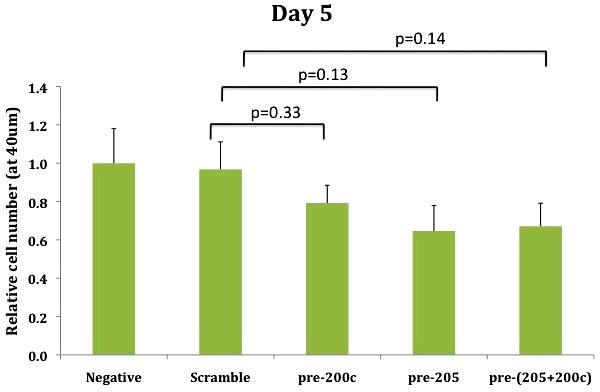


**B**


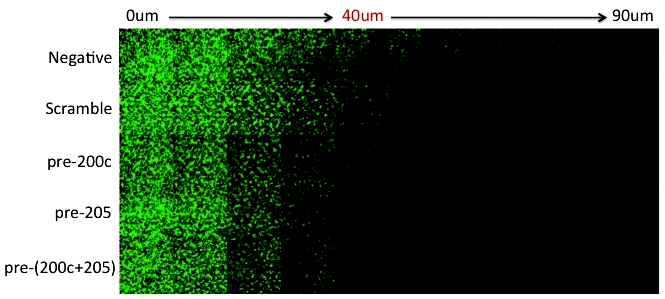


**Supplementary Figure 5** Ectopic expression of miR-200c and miR-205 in cultured melanoma cells fails to cause reduced invasive ability. A375 melanoma cells were transfected with miR-200c and miR-205 precursors, or a scrambled control miRNA and invasion in a transwell migration assay was determined. (**A**) Cells migrating through matrigel were quantified after 5 days at the 40 μm layer. The relative cell number was determined as the ratio of the cell number at the 40 μm layer/ the cell number at the origin (0 μm layer). Results represent average ratios (± SEM) of six microscope fields for each group, normalized to the negative control group. (**B**) Representative stacked confocal images from the origin (0 μm layer) to the tenth layer (90 μm). Each layer is 10 μm thick. The 40 μm-layer indicated in red was used to quantify relative cell number.

**Supplementary Table 1**

| **Sample type** | **Code** | **Description** |
| --- | --- | --- |
| Cells |  |  |
| Melanocytes | H1  H4 | Hermes 1  Hermes 4a |
| EDMEL3 melanoma series | ED  EP2  ES1  ES2  ES4  ES5  ET1  ET2 | Passage 10  Passage 2  Subclone 1  Subclone 2  Subclone 4  Subclone 5  Xenograft 1  Xenograft 2 |
| Other melanoma cells | A3  C3  G3  HB  WM | A375  C32  G361  HBL  WM115 |
| FFPE |  |  |
| Benign naevus | N plus # | 11 samples |
| Primary melanoma | RG1-10  RG11-20 | Non-recurrent  Recurrent |
| Metastatic melanoma | M or ME plus # | 21 samples |

**Supplementary Table 1** Coding details of the cell and FFPE samples on the array. Primary and metastatic melanomas were obtained from different patients.

**Supplementary Table 2** Differentially expressed microRNAs in benign naevi, primary and metastatic melanoma FFPE tissues. (**A**) Primary melanoma versus naevus. (**B**) Metastatic melanoma versus naevus; (**C**) Metastatic versus primary melanoma. The tables list all the differentially expressed miRNAs with an adjusted *P* value < 0.05, ranked by adjusted *P* value. Log_2_ FC is the log_2_ fold expression difference. In X vs. Y comparison, when Y>X, log_2_ FC is negative. Average Intensity is the average signal intensity for the particular miRNA of all samples on the array.

**(A) Comparison between primary melanomas** and naevi

| **No** | **miR** | **Adjusted *P*** | ***P* value** | **Log**_2_ **FC** | **Log**_2_ **Average**  **Intensity** |
| --- | --- | --- | --- | --- | --- |
| 1 | solexa-2580-353 | 3.52E-13 | 4.08E-16 | -2.67 | 8.51 |
| 2 | solexa-8048-104 | 1.45E-12 | 4.51E-15 | 3.72 | 13.27 |
| 3 | hsa-miR-603 | 1.45E-12 | 5.06E-15 | 2.79 | 11.71 |
| 4 | solexa-2683-338 | 5.33E-12 | 2.47E-14 | 4.05 | 11.83 |
| 5 | HS_29 | 9.76E-11 | 5.66E-13 | 3.86 | 12.90 |
| 6 | hsa-miR-663b | 7.04E-10 | 4.90E-12 | 3.43 | 12.22 |
| 7 | hsa-miR-1826 | 8.69E-10 | 7.06E-12 | 2.23 | 13.60 |
| 8 | hsa-miR-200b* | 1.26E-09 | 1.17E-11 | -3.52 | 9.32 |
| 9 | hsa-miR-1308 | 1.69E-09 | 1.76E-11 | 0.81 | 14.56 |
| 10 | solexa-578-1915 | 2.47E-09 | 3.10E-11 | 3.50 | 11.78 |
| 11 | hsa-miR-183 | 2.47E-09 | 3.15E-11 | -3.06 | 8.96 |
| 12 | hsa-miR-149 | 5.13E-07 | 7.15E-09 | -2.41 | 9.12 |
| 13 | hsa-miR-200a | 1.55E-06 | 2.34E-08 | -2.79 | 9.52 |
| 14 | hsa-miR-205 | 2.49E-06 | 4.05E-08 | -2.44 | 11.34 |
| 15 | HS_22.1 | 4.35E-06 | 7.57E-08 | 2.28 | 13.07 |
| 16 | hsa-miR-675 | 4.87E-06 | 9.04E-08 | 3.06 | 11.11 |
| 17 | hsa-miR-99a | 6.97E-06 | 1.39E-07 | -2.03 | 11.84 |
| 18 | hsa-miR-200b | 6.97E-06 | 1.46E-07 | -3.04 | 11.83 |
| 19 | HS_33 | 7.20E-06 | 1.59E-07 | 1.87 | 10.44 |
| 20 | hsa-miR-455-5p | 8.39E-06 | 1.95E-07 | -2.45 | 9.60 |
| 21 | HS_263.1 | 1.10E-05 | 2.68E-07 | 1.12 | 13.78 |
| 22 | hsa-miR-182 | 1.76E-05 | 4.48E-07 | -2.82 | 9.69 |
| 23 | hsa-miR-203 | 2.91E-05 | 7.75E-07 | -2.62 | 10.88 |
| 24 | hsa-miR-494 | 4.99E-05 | 1.39E-06 | 2.91 | 11.36 |
| 25 | hsa-miR-145 | 8.96E-05 | 2.60E-06 | -0.91 | 13.85 |
| 26 | HS_35 | 1.42E-04 | 4.27E-06 | 2.76 | 9.23 |
| 27 | hsa-miR-455-3p | 1.93E-04 | 6.03E-06 | -2.13 | 12.51 |
| 28 | hsa-miR-20b | 2.55E-04 | 8.28E-06 | 2.43 | 11.16 |
| 29 | hsa-miR-95 | 3.54E-04 | 1.19E-05 | 1.40 | 13.50 |
| 30 | hsa-miR-888 | 3.97E-04 | 1.38E-05 | -1.86 | 8.85 |
| 31 | hsa-miR-891b | 4.72E-04 | 1.70E-05 | -1.34 | 8.12 |
| 32 | hsa-miR-141 | 5.14E-04 | 1.91E-05 | -2.16 | 10.03 |
| 33 | hsa-miR-519e* | 6.76E-04 | 2.59E-05 | 1.88 | 8.92 |
| 34 | hsa-miR-198 | 7.98E-04 | 3.15E-05 | 2.45 | 9.09 |
| 35 | hsa-miR-195 | 9.29E-04 | 3.77E-05 | -1.80 | 12.92 |
| 36 | hsa-miR-923 | 9.86E-04 | 4.16E-05 | 0.88 | 14.11 |
| 37 | hsa-miR-224 | 9.86E-04 | 4.23E-05 | -2.54 | 10.13 |
| 38 | hsa-miR-378 | 1.02E-03 | 4.50E-05 | -2.02 | 12.21 |
| 39 | HS_43.1 | 1.30E-03 | 5.86E-05 | 1.09 | 8.92 |
| 40 | hsa-miR-1285 | 1.32E-03 | 6.12E-05 | 2.65 | 12.37 |
| 41 | hsa-miR-1273 | 1.41E-03 | 6.72E-05 | 2.89 | 11.67 |
| 42 | HS_143 | 1.48E-03 | 7.21E-05 | 2.05 | 10.69 |
| 43 | hsa-miR-154 | 1.98E-03 | 9.87E-05 | -1.63 | 8.58 |
| 44 | hsa-miR-200c | 2.12E-03 | 1.08E-04 | -1.41 | 13.04 |
| 45 | hsa-miR-30c-1* | 2.55E-03 | 1.33E-04 | 1.76 | 13.84 |
| 46 | hsa-miR-876-3p | 2.68E-03 | 1.43E-04 | -1.90 | 9.48 |
| 47 | solexa-3927-221 | 2.97E-03 | 1.62E-04 | 0.58 | 14.29 |
| 48 | hsa-miR-139-5p | 3.85E-03 | 2.14E-04 | -1.79 | 10.41 |
| 49 | hsa-miR-1274a | 4.02E-03 | 2.34E-04 | 0.47 | 14.65 |
| 50 | hsa-miR-429 | 4.02E-03 | 2.34E-04 | -1.75 | 9.14 |
| 51 | hsa-miR-1248 | 4.02E-03 | 2.38E-04 | 2.36 | 10.94 |
| 52 | hsa-miR-497 | 4.65E-03 | 2.81E-04 | -1.78 | 10.14 |
| 53 | hsa-miR-29b-2* | 5.05E-03 | 3.11E-04 | -1.07 | 8.39 |
| 54 | HS_78 | 5.06E-03 | 3.20E-04 | 0.99 | 9.33 |
| 55 | HS_40 | 5.06E-03 | 3.29E-04 | -0.87 | 9.59 |
| 56 | hsa-miR-125b | 5.06E-03 | 3.29E-04 | -0.59 | 14.39 |
| 57 | hsa-miR-885-5p | 5.42E-03 | 3.58E-04 | -1.07 | 8.25 |
| 58 | hsa-miR-27b* | 5.53E-03 | 3.72E-04 | -1.18 | 9.94 |
| 59 | hsa-miR-204 | 5.58E-03 | 3.82E-04 | -1.52 | 11.18 |
| 60 | HS_100 | 5.69E-03 | 3.96E-04 | 0.26 | 14.55 |
| 61 | hsa-miR-140-3p | 6.57E-03 | 4.69E-04 | -1.19 | 12.83 |
| 62 | hsa-miR-1300 | 6.57E-03 | 4.73E-04 | 1.51 | 9.77 |
| 63 | hsa-miR-27a* | 7.44E-03 | 5.44E-04 | -1.09 | 9.41 |
| 64 | hsa-miR-1249 | 8.38E-03 | 6.22E-04 | -1.59 | 11.76 |
| 65 | hsa-miR-431 | 8.51E-03 | 6.41E-04 | -1.15 | 8.85 |
| 66 | hsa-miR-1290 | 8.66E-03 | 6.63E-04 | -1.82 | 10.12 |
| 67 | hsa-miR-189:9.1 | 8.71E-03 | 6.77E-04 | -1.14 | 10.82 |
| 68 | hsa-miR-365 | 8.91E-03 | 7.03E-04 | -1.15 | 11.23 |
| 69 | solexa-2952-306 | 1.12E-02 | 9.02E-04 | 0.32 | 14.28 |
| 70 | solexa-5620-151 | 1.12E-02 | 9.07E-04 | 1.67 | 9.22 |
| 71 | hsa-miR-567 | 1.16E-02 | 9.67E-04 | -0.52 | 8.39 |
| 72 | solexa-826-1288 | 1.16E-02 | 9.72E-04 | 1.90 | 10.26 |
| 73 | solexa-15-44487 | 1.34E-02 | 1.14E-03 | 0.92 | 10.32 |
| 74 | solexa-8211-102 | 1.36E-02 | 1.17E-03 | 1.47 | 13.46 |
| 75 | solexa-555-1991 | 1.37E-02 | 1.19E-03 | 0.74 | 13.34 |
| 76 | hsa-miR-127-3p | 1.84E-02 | 1.63E-03 | -1.06 | 8.74 |
| 77 | hsa-miR-639 | 1.84E-02 | 1.64E-03 | 1.38 | 8.60 |
| 78 | hsa-miR-565:9.1 | 1.88E-02 | 1.70E-03 | 0.63 | 14.07 |
| 79 | HS_112 | 2.16E-02 | 1.98E-03 | 1.03 | 8.29 |
| 80 | hsa-miR-576-3p | 2.19E-02 | 2.03E-03 | 1.12 | 9.11 |
| 81 | HS_241.1 | 2.34E-02 | 2.20E-03 | 1.47 | 10.45 |
| 82 | hsa-miR-125b-2* | 2.38E-02 | 2.26E-03 | -1.25 | 8.51 |
| 83 | hsa-miR-199a*:9.1 | 2.43E-02 | 2.34E-03 | -0.89 | 13.36 |
| 84 | hsa-miR-9* | 2.67E-02 | 2.60E-03 | 1.33 | 10.89 |
| 85 | HS_126 | 2.82E-02 | 2.78E-03 | 0.96 | 8.61 |
| 86 | hsa-miR-1228* | 2.89E-02 | 2.89E-03 | 0.48 | 13.89 |
| 87 | solexa-4793-177 | 3.15E-02 | 3.21E-03 | 1.16 | 12.26 |
| 88 | hsa-miR-140-5p | 3.15E-02 | 3.22E-03 | -1.53 | 10.87 |
| 89 | HS_186 | 3.21E-02 | 3.31E-03 | -0.82 | 9.65 |
| 90 | hsa-miR-25* | 3.29E-02 | 3.43E-03 | 0.80 | 9.97 |
| 91 | hsa-miR-9 | 3.59E-02 | 3.85E-03 | 1.87 | 10.76 |
| 92 | hsa-miR-218 | 3.59E-02 | 3.85E-03 | -1.49 | 10.61 |
| 93 | hsa-miR-342-5p | 3.59E-02 | 3.87E-03 | -1.25 | 11.40 |
| 94 | hsa-miR-591 | 3.86E-02 | 4.24E-03 | 0.81 | 9.49 |
| 95 | HS_196.1 | 3.86E-02 | 4.26E-03 | 1.11 | 8.55 |
| 96 | HS_188 | 4.15E-02 | 4.70E-03 | 1.42 | 10.95 |
| 97 | hsa-miR-100 | 4.15E-02 | 4.71E-03 | -0.86 | 13.64 |
| 98 | hsa-miR-335 | 4.15E-02 | 4.72E-03 | 0.90 | 11.76 |
| 99 | hsa-miR-196a* | 4.29E-02 | 4.95E-03 | 0.83 | 8.34 |
| 100 | hsa-miR-28-3p | 4.29E-02 | 4.97E-03 | -1.03 | 12.42 |
| 101 | HS_31.1 | 4.40E-02 | 5.19E-03 | 1.05 | 9.40 |
| 102 | hsa-miR-181a-2* | 4.40E-02 | 5.20E-03 | -1.44 | 10.97 |
| 103 | hsa-miR-139-3p | 4.60E-02 | 5.52E-03 | -0.69 | 8.42 |
| 104 | hsa-miR-744* | 4.60E-02 | 5.55E-03 | -0.36 | 8.34 |
| 105 | hsa-miR-921 | 4.91E-02 | 6.10E-03 | -0.73 | 8.46 |
| 106 | hsa-miR-875-5p | 4.91E-02 | 6.11E-03 | -0.56 | 8.87 |
| 107 | hsa-miR-664 | 4.91E-02 | 6.11E-03 | 0.65 | 13.49 |
| 108 | hsa-miR-767-5p | 4.91E-02 | 6.19E-03 | 1.21 | 10.43 |
| 109 | HS_116 | 4.91E-02 | 6.30E-03 | 1.66 | 10.65 |
| 111 | hsa-miR-147b | 4.91E-02 | 6.33E-03 | -0.99 | 9.06 |
| 112 | hsa-miR-335* | 4.91E-02 | 6.35E-03 | -1.13 | 8.90 |
| 113 | hsa-miR-873 | 4.91E-02 | 6.40E-03 | -1.31 | 9.33 |

**(B) Comparison between metastatic melanomas** and naevi

| **No** | **miR** | **Adjusted *P*** | ***P* value** | **Log**_2_ **FC** | **Log**_2_ **Average**  **Intensity** |
| --- | --- | --- | --- | --- | --- |
| 1 | hsa-miR-205 | 3.88E-16 | 4.50E-19 | -5.23 | 11.34 |
| 2 | solexa-2580-353 | 8.42E-16 | 1.95E-18 | -3.03 | 8.51 |
| 3 | hsa-miR-203 | 2.06E-15 | 7.18E-18 | -6.00 | 10.88 |
| 4 | hsa-miR-183 | 6.41E-11 | 2.98E-13 | -3.52 | 8.96 |
| 5 | solexa-2683-338 | 1.19E-10 | 6.93E-13 | 3.65 | 11.83 |
| 6 | hsa-miR-200b* | 1.27E-10 | 9.28E-13 | -3.78 | 9.32 |
| 7 | hsa-miR-200c | 1.27E-10 | 1.03E-12 | -3.13 | 13.04 |
| 8 | hsa-miR-603 | 1.26E-09 | 1.17E-11 | 2.20 | 11.71 |
| 9 | solexa-8048-104 | 4.64E-09 | 4.85E-11 | 2.78 | 13.27 |
| 10 | hsa-miR-1308 | 2.38E-08 | 2.76E-10 | 0.73 | 14.56 |
| 11 | hsa-miR-200b | 8.86E-08 | 1.14E-09 | -3.67 | 11.83 |
| 12 | hsa-miR-200a | 8.86E-08 | 1.23E-09 | -3.11 | 9.52 |
| 13 | hsa-miR-663b | 9.07E-08 | 1.37E-09 | 2.81 | 12.22 |
| 14 | HS_29 | 1.03E-07 | 1.67E-09 | 2.93 | 12.90 |
| 15 | solexa-578-1915 | 4.56E-07 | 7.94E-09 | 2.84 | 11.78 |
| 16 | hsa-miR-1826 | 6.26E-07 | 1.16E-08 | 1.70 | 13.60 |
| 17 | hsa-miR-141 | 2.24E-06 | 4.42E-08 | -2.92 | 10.03 |
| 18 | hsa-miR-149 | 4.38E-06 | 9.14E-08 | -2.15 | 9.12 |
| 19 | solexa-555-1991 | 9.09E-06 | 2.00E-07 | 1.28 | 13.34 |
| 20 | hsa-miR-95 | 9.86E-06 | 2.29E-07 | 1.71 | 13.50 |
| 21 | hsa-miR-429 | 1.91E-05 | 4.66E-07 | -2.53 | 9.14 |
| 22 | hsa-miR-21* | 2.42E-05 | 6.17E-07 | 2.12 | 9.86 |
| 23 | hsa-miR-224 | 3.06E-05 | 8.23E-07 | -3.16 | 10.13 |
| 24 | hsa-miR-182 | 3.06E-05 | 8.52E-07 | -2.71 | 9.69 |
| 25 | hsa-miR-142-5p | 1.02E-04 | 2.96E-06 | 2.26 | 9.50 |
| 26 | hsa-miR-923 | 2.11E-04 | 6.36E-06 | 0.98 | 14.11 |
| 27 | hsa-miR-20b | 2.26E-04 | 7.09E-06 | 2.43 | 11.16 |
| 28 | hsa-miR-675 | 2.90E-04 | 9.43E-06 | 2.40 | 11.11 |
| 29 | hsa-miR-891b | 4.47E-04 | 1.54E-05 | -1.33 | 8.12 |
| 30 | hsa-miR-888 | 4.47E-04 | 1.56E-05 | -1.83 | 8.85 |
| 31 | hsa-miR-9 | 5.51E-04 | 1.98E-05 | 2.88 | 10.76 |
| 32 | hsa-miR-455-5p | 6.04E-04 | 2.24E-05 | -1.89 | 9.60 |
| 33 | hsa-let-7c | 8.37E-04 | 3.21E-05 | -0.90 | 13.81 |
| 34 | hsa-miR-9* | 1.02E-03 | 4.00E-05 | 1.88 | 10.89 |
| 35 | hsa-miR-155 | 1.02E-03 | 4.14E-05 | 1.31 | 13.28 |
| 36 | HS_100 | 1.46E-03 | 6.21E-05 | 0.29 | 14.55 |
| 37 | hsa-miR-455-3p | 1.46E-03 | 6.44E-05 | -1.82 | 12.51 |
| 38 | hsa-miR-873 | 1.46E-03 | 6.46E-05 | -1.99 | 9.33 |
| 39 | hsa-miR-876-3p | 1.57E-03 | 7.12E-05 | -1.98 | 9.48 |
| 40 | hsa-miR-139-5p | 1.92E-03 | 8.90E-05 | -1.89 | 10.41 |
| 41 | hsa-miR-548d-5p | 2.01E-03 | 9.58E-05 | -1.08 | 9.71 |
| 42 | hsa-miR-1300 | 2.29E-03 | 1.12E-04 | 1.67 | 9.77 |
| 43 | hsa-miR-204 | 2.58E-03 | 1.29E-04 | -1.64 | 11.18 |
| 44 | HS_264.1 | 2.85E-03 | 1.45E-04 | 0.76 | 8.56 |
| 45 | hsa-miR-567 | 3.21E-03 | 1.67E-04 | -0.60 | 8.39 |
| 46 | solexa-3927-221 | 3.42E-03 | 1.82E-04 | 0.57 | 14.29 |
| 47 | hsa-miR-1274a | 3.45E-03 | 1.89E-04 | 0.47 | 14.65 |
| 48 | hsa-miR-365 | 3.45E-03 | 1.92E-04 | -1.27 | 11.23 |
| 49 | hsa-miR-1321 | 4.05E-03 | 2.30E-04 | 0.45 | 8.41 |
| 50 | HS_108.1 | 4.26E-03 | 2.47E-04 | -2.18 | 11.17 |
| 51 | hsa-miR-1248 | 4.67E-03 | 2.77E-04 | 2.32 | 10.94 |
| 52 | solexa-826-1288 | 4.71E-03 | 2.84E-04 | 2.09 | 10.26 |
| 53 | hsa-miR-193b | 4.96E-03 | 3.05E-04 | -1.62 | 12.93 |
| 54 | solexa-15-44487 | 6.83E-03 | 4.28E-04 | 1.00 | 10.32 |
| 55 | HS_33 | 7.55E-03 | 4.87E-04 | 1.14 | 10.44 |
| 56 | HS_114 | 7.55E-03 | 4.90E-04 | -0.62 | 8.94 |
| 57 | hsa-miR-1290 | 8.11E-03 | 5.51E-04 | -1.84 | 10.12 |
| 58 | hsa-miR-142-3p | 8.11E-03 | 5.52E-04 | 1.55 | 12.74 |
| 59 | hsa-miR-508-3p | 8.11E-03 | 5.55E-04 | -2.56 | 12.66 |
| 60 | hsa-miR-99a | 8.14E-03 | 5.67E-04 | -1.21 | 11.84 |
| 61 | hsa-miR-10a* | 8.14E-03 | 5.76E-04 | 1.32 | 10.40 |
| 62 | hsa-miR-211 | 8.75E-03 | 6.29E-04 | -2.37 | 12.93 |
| 63 | hsa-miR-23b* | 9.27E-03 | 6.77E-04 | -1.40 | 8.93 |
| 64 | HS_75.1 | 9.70E-03 | 7.20E-04 | -1.07 | 8.69 |
| 65 | hsa-miR-767-5p | 9.91E-03 | 7.47E-04 | 1.51 | 10.43 |
| 66 | HS_93 | 1.02E-02 | 7.84E-04 | 0.77 | 8.81 |
| 67 | HS_43.1 | 1.02E-02 | 7.94E-04 | 0.88 | 8.92 |
| 68 | HS_78 | 1.02E-02 | 8.07E-04 | 0.91 | 9.33 |
| 69 | hsa-miR-449a | 1.02E-02 | 8.20E-04 | -1.69 | 8.93 |
| 70 | hsa-miR-513:9.1 | 1.16E-02 | 9.46E-04 | -2.10 | 9.97 |
| 71 | hsa-miR-509-3p | 1.24E-02 | 1.02E-03 | -2.31 | 13.39 |
| 72 | hsa-miR-27a* | 1.24E-02 | 1.04E-03 | -1.02 | 9.41 |
| 73 | HS_194 | 1.25E-02 | 1.06E-03 | -1.00 | 8.20 |
| 74 | hsa-miR-154 | 1.30E-02 | 1.12E-03 | -1.32 | 8.58 |
| 75 | hsa-miR-506 | 1.32E-02 | 1.15E-03 | -2.39 | 12.17 |
| 76 | hsa-miR-542-3p | 1.37E-02 | 1.21E-03 | 1.12 | 9.52 |
| 77 | hsa-miR-137 | 1.38E-02 | 1.23E-03 | 0.95 | 10.32 |
| 78 | HS_263.1 | 1.39E-02 | 1.26E-03 | 0.64 | 13.78 |
| 79 | hsa-miR-618 | 1.41E-02 | 1.29E-03 | 0.76 | 9.33 |
| 80 | HS_40 | 1.46E-02 | 1.35E-03 | -0.76 | 9.59 |
| 81 | HS_22.1 | 1.57E-02 | 1.47E-03 | 1.21 | 13.07 |
| 82 | hsa-miR-146b-3p | 1.62E-02 | 1.54E-03 | 0.50 | 8.71 |
| 83 | hsa-miR-105 | 1.63E-02 | 1.57E-03 | 1.14 | 10.81 |
| 84 | hsa-miR-189:9.1 | 1.70E-02 | 1.66E-03 | -1.04 | 10.82 |
| 85 | hsa-miR-513a-5p | 1.95E-02 | 1.92E-03 | -1.55 | 11.46 |
| 86 | hsa-miR-196a | 2.03E-02 | 2.03E-03 | 1.43 | 13.41 |
| 87 | hsa-miR-1249 | 2.11E-02 | 2.14E-03 | -1.40 | 11.76 |
| 88 | hsa-miR-27b | 2.11E-02 | 2.16E-03 | -0.86 | 13.49 |
| 89 | hsa-miR-664 | 2.79E-02 | 2.88E-03 | 0.71 | 13.49 |
| 90 | hsa-miR-885-5p | 3.13E-02 | 3.28E-03 | -0.86 | 8.25 |
| 91 | hsa-miR-1202 | 3.13E-02 | 3.31E-03 | 0.44 | 8.40 |
| 92 | HS_241.1 | 3.24E-02 | 3.46E-03 | 1.39 | 10.45 |
| 93 | hsa-miR-16-1* | 3.38E-02 | 3.65E-03 | 0.83 | 10.12 |
| 94 | hsa-miR-801:9.1 | 3.81E-02 | 4.15E-03 | 0.70 | 8.69 |
| 95 | hsa-miR-513c | 4.08E-02 | 4.50E-03 | -1.58 | 9.62 |
| 96 | hsa-miR-199a*:9.1 | 4.17E-02 | 4.64E-03 | -0.81 | 13.36 |
| 97 | hsa-miR-147b | 4.94E-02 | 5.56E-03 | -0.99 | 9.06 |

**(C) Comparison between metastatic and primary melanomas**

| **No** | **miR** | **Adjusted *P*** | ***P* value** | **Log**_2_ **FC** | **Log**_2_ **Average**  **Intensity** |
| --- | --- | --- | --- | --- | --- |
| 1 | hsa-miR-205 | 4.42E-09 | 6.64E-12 | -2.79 | 11.34 |
| 2 | hsa-miR-203 | 4.42E-09 | 1.03E-11 | -3.38 | 10.88 |
| 3 | hsa-miR-145 | 4.16E-06 | 1.45E-08 | 0.97 | 13.85 |
| 4 | hsa-miR-200c | 2.71E-05 | 1.26E-07 | -1.72 | 13.04 |
| 5 | hsa-miR-142-5p | 7.14E-04 | 4.14E-06 | 1.86 | 9.50 |
| 6 | hsa-miR-519e* | 2.11E-03 | 1.47E-05 | -1.62 | 8.92 |
| 7 | hsa-miR-198 | 5.06E-03 | 4.11E-05 | -2.00 | 9.09 |
| 8 | hsa-miR-21* | 1.22E-02 | 1.19E-04 | 1.30 | 9.86 |
| 9 | hsa-miR-452 | 1.22E-02 | 1.27E-04 | -1.24 | 9.52 |
| 10 | hsa-miR-1296 | 1.96E-02 | 2.28E-04 | 1.77 | 9.60 |
| 11 | hsa-miR-509-3p | 2.11E-02 | 2.76E-04 | -2.17 | 13.39 |
| 12 | hsa-miR-150 | 2.11E-02 | 3.10E-04 | 1.01 | 14.05 |
| 13 | hsa-miR-19a | 2.11E-02 | 3.19E-04 | 1.41 | 10.40 |
| 14 | hsa-miR-22 | 3.42E-02 | 5.55E-04 | 0.74 | 12.69 |
| 15 | hsa-miR-506 | 4.46E-02 | 7.76E-04 | -2.09 | 12.17 |
| 16 | hsa-miR-542-5p | 4.49E-02 | 8.77E-04 | 1.53 | 9.70 |
| 17 | HS_22.1 | 4.49E-02 | 8.95E-04 | -1.07 | 13.07 |
| 18 | HS_143 | 4.49E-02 | 9.39E-04 | -1.39 | 10.69 |
| 19 | hsa-miR-558 | 4.79E-02 | 1.15E-03 | 1.31 | 9.80 |
| 20 | hsa-miR-29c | 4.79E-02 | 1.22E-03 | 1.14 | 13.03 |
| 21 | hsa-miR-28-3p | 4.79E-02 | 1.27E-03 | 0.99 | 12.42 |
| 22 | HS_132.1 | 4.79E-02 | 1.32E-03 | -1.28 | 9.60 |
| 23 | hsa-miR-514 | 4.79E-02 | 1.32E-03 | -2.47 | 12.60 |
| 24 | hsa-miR-494 | 4.79E-02 | 1.33E-03 | -1.51 | 11.36 |
| 25 | hsa-miR-140-3p | 4.79E-02 | 1.39E-03 | 0.89 | 12.83 |

**Supplementary Table 3** Down regulation of miR-20b in A375 melanoma cells transfected with a miR-20b inhibitor. A375 melanoma cells were transfected with miR-20b inhibitor miRNA, or a scrambled control miRNA. Mean relative expression levels of miR-20b (± SEM), relative to miR-92 and normalized to the mean of the negative control from three independent experiments, are shown for the three days following transfection.

|  | **Negative** | **Scramble** | **anti-20b** |
| --- | --- | --- | --- |
| **24hr** | 1.00±0.14 | 1.21±0.30 | 0.23±0.04 |
| **48hr** | 1.00±0.20 | 0.91±0.06 | 0.45±0.06 |
| **72hr** | 1.00±0.20 | 0.91±0.28 | 0.96±0.19 |
